# Efficacy and Safety of Vilaprisan in the Treatment of Uterine Fibroids: Data from ASTEROID 5, a Phase 3 Multicenter Randomized Controlled Trial

**DOI:** 10.3390/jcm15093246

**Published:** 2026-04-24

**Authors:** K. Gemzell-Danielsson, C.-H. Cho, P. Vadász, R. Wenzl, L. Dong, T. Faustmann, E. Groettrup-Wolfers, K. Laapas, S. Parke, C. Haberland, C. Seitz

**Affiliations:** 1 Department of Women’s and Children’s Health, Karolinska Institutet, 171 77 Stockholm, Sweden; kristina.gemzell@ki.se; 2 Division of Gynecology and Reproductive Medicine, Karolinska University Hospital, 171 76 Stockholm, Sweden; 3 Department of Obstetrics and Gynecology, School of Medicine, Keimyung University, Daegu 42601, Republic of Korea; chcho28@gmail.com; 4 Department of Obstetrics and Gynecology, Selye János Hospital, 2900 Komárom, Hungary; info@drvadaszpeter.hu; 5 Department of Obstetrics and Gynecology, Medical University of Vienna, 1090 Vienna, Austria; rene.wenzl@meduniwien.ac.at; 6 Bayer AG, 13353 Berlin, Germany; dong_phdly@t-online.de (L.D.); faustmannt@gedeonrichter.com (T.F.); esther.groettrup-wolfers@bayer.com (E.G.-W.); kafra21@web.de (S.P.); claudia.haberland@bayer.com (C.H.); 7 Bayer Oy, 02100 Espoo, Finland; kaisa.laapas@bayer.com; 8 Institute of Clinical Pharmacology and Toxicology, Charité–Universitätsmedizin Berlin, 10117 Berlin, Germany

**Keywords:** amenorrhea, heavy menstrual bleeding, selective progesterone receptor modulator, uterine fibroids, vilaprisan

## Abstract

**Background/Objectives**: Vilaprisan and ulipristal acetate (UPA) have demonstrated efficacy in treating uterine fibroids (UFs). However, a direct comparison of vilaprisan and UPA has been restricted, to date, to a small phase 2 study. Here, we compare the efficacy of vilaprisan and UPA in reducing heavy menstrual bleeding and inducing amenorrhea in women with symptomatic UFs. **Methods**: ASTEROID 5 (NCT03240523) was planned as a randomized, active-controlled, multicenter phase 3 study comparing three vilaprisan 2 mg/day regimens against the approved regimen of UPA 5 mg/day for the treatment of symptomatic UFs. Participants were initially randomized 1:1:1:1 to one of four treatment arms: VPR-3/1 (vilaprisan for a 3-month treatment period [TP] followed by one menstrual bleeding episode); VPR-6/2 (vilaprisan for a 6-month TP followed by two menstrual bleeding episodes); VPR-3/2 (vilaprisan plus matching UPA placebo for a 3-month TP followed by two menstrual bleeding episodes); and UPA-3/2 (UPA plus matching vilaprisan placebo for a 3-month TP followed by two menstrual bleeding episodes). **Results**: Treatment was received as planned by 271 (95.4%), 266 (94.0%), 90 (90.0%) and 89 (89.9%) women in the VPR-3/1, VPR-6/2, VPR-3/2, and UPA-3/2 groups, respectively; 109 women in the VPR-3/1 (*n* = 44, 15.5%) and VPR-6/2 (*n* = 65, 23.0%) groups and none in the VPR-3/2 and UPA-3/2 groups completed treatment. Vilaprisan (total VPR group) demonstrated non-inferiority but not superiority versus UPA in inducing amenorrhea (82.9% [520/627] vs. 74.2% [66/89]; difference: 8.8% [95% confidence interval: −0.78, 18.34]; *p* = 0.0553), whereas vilaprisan (VPR-3/1 arm) showed superiority versus UPA in reducing total menstrual blood loss (least squares mean total MBL: 44.2 mL vs. 80.3 mL; difference: −36.1 mL; *p* = 0.0010). **Conclusions**: Vilaprisan (VPR-3/1 regimen) was superior to UPA in reducing total MBL, and it was non-inferior (total VPR group) to UPA in inducing amenorrhea.

## 1. Introduction

Uterine fibroids (UFs) are the most common benign pelvic tumors in women, although their prevalence may be underestimated as many women are asymptomatic and are often diagnosed incidentally [1]. However, there is evidence that 25% of women with UFs develop severe symptoms that will require treatment and have a negative impact on their quality of life (QoL) [2,3]. The most common symptom associated with UFs is heavy menstrual bleeding (HMB; commonly defined as >80 mL menstrual blood loss [MBL] per cycle), which occurs in up to 40% of women and is often associated with iron deficiency anemia [4,5]. Other common symptoms of UFs include lower back pain and pelvic discomfort, which are influenced by the location and size of the UFs [3,6]. UFs may also be associated with reproductive problems, including infertility, recurrent pregnancy loss, and adverse obstetric outcomes [6]. Due to their high prevalence, UFs pose a substantial economic burden on healthcare systems worldwide [7].

Although hysterectomy can permanently remove UFs, this is at the expense of the ability to become pregnant; uterus-sparing procedures such as a myomectomy are also possible, but these carry a significant risk of UF recurrence [7]. Clinical trials have indicated that selective progesterone receptor modulators (SPRMs), such as ulipristal acetate (UPA) [8,9,10,11,12], and oral gonadotropin-releasing hormone (GnRH) antagonists, such as elagolix [13,14,15] and relugolix [16,17], may be used for the treatment of UFs and associated HMB. However, post-authorization reports of liver injury [18] led to UPA being withdrawn from the Canadian market [19] and its use in Europe being restricted to premenopausal women who are unsuitable candidates for surgery [20]. In the United States, UPA has not received approval for the treatment of UFs. Furthermore, although GnRH antagonists have shown efficacy in the treatment of HMB, their effects on fibroid volume are generally less marked than those reported for UPA [13,14,15,16], and their hypoestrogenic effects mandate the use of concomitant hormonal add-back therapy, which in turn may limit efficacy due to potentially increasing uterine volume and bleeding [21,22,23,24].

Vilaprisan (VPR) is a novel, highly potent SPRM that has been assessed in women for the treatment of symptomatic UFs and endometriosis [25]. It has a five-fold greater antiprogestogenic potency than UPA in vivo [26]. Vilaprisan is well tolerated and has a favorable pharmacokinetic and pharmacodynamic profile, as demonstrated by the ASTEROID clinical trial program [25,27,28,29,30]. Furthermore, vilaprisan has been shown to control bleeding, induce amenorrhea, reduce fibroid volume, and relieve the symptoms associated with UFs [27,29,30,31,32].

In the phase 3 ASTEROID 5 randomized clinical trial, the efficacy of vilaprisan and UPA in reducing HMB and inducing amenorrhea in women with symptomatic UFs was compared. The vilaprisan clinical program, including this phase 3 trial, was placed on hold in December 2018 based on the findings of animal carcinogenicity studies. Consequently, women who had already initiated treatment before this date were instructed to discontinue the study drug after completing their ongoing treatment period (TP) and were followed up for safety. This paper presents the findings from the treatment and follow-up of these women, with a primary focus on assessing the impact of vilaprisan on HMB compared with UPA.

## 2. Materials and Methods

### 2.1. Study Design

ASTEROID 5 (ClinicalTrials.gov: NCT03240523) was planned as a randomized, multicenter phase 3 study, with elements of a double-blind, double-dummy, and open-label design. The primary objective of the study was to compare the efficacy of vilaprisan and UPA in women with UFs. The secondary objective was to evaluate the efficacy and safety of different vilaprisan treatment regimens.

The screening period was kept to a maximum of 90 days, and the overall treatment phase comprised a sequence of TPs during which women received study medication, separated by treatment breaks to allow for a specific number of menstrual bleeding episodes.

Eligible women were initially randomized 1:1:1:1 to one of four treatment groups: vilaprisan administered for four TPs of 12 weeks (i.e., 3 months, 3 × 28 days) with one menstrual bleeding episode between TPs (VPR-3/1); vilaprisan administered for two TPs of 24 weeks (i.e., 6 months, 6 × 28 days) with two bleeding episodes between TPs (VPR-6/2); vilaprisan with matching placebo to UPA administered for up to two TPs of 12 weeks (i.e., 3 months, 3 × 28 days) with two bleeding episodes between TPs (VPR-3/2); and UPA with matching placebo to vilaprisan administered for up to two TPs of 12 weeks (i.e., 3 months, 3 × 28 days) with two bleeding episodes between TPs (UPA-3/2). Vilaprisan was given at a dose of 2 mg/day and UPA at the approved dose of 5 mg/day. The different treatment regimens implemented in the VPR-3/1 and VPR-6/2 groups meant that they had to be open-label. However, the VPR-3/2 and UPA-3/2 groups were blinded against each other (double-blind and double-dummy).

Study recruitment and treatment started in July 2017. Randomization to the blinded groups ceased in February 2018 to comply with the outcome of the UPA safety assessment conducted by the Pharmacovigilance Risk Assessment Committee (PRAC), which found an increased risk of liver injury in women with UFs receiving UPA, and subsequently limited its use to a small subset of patients [33]. Women previously randomized to the VPR-3/2 and UPA-3/2 groups were offered to continue in the study on open-label VPR-3/2 treatment for up to 1 year (remaining blinded to the treatment received prior to the amendment). The study design following the protocol amendment due to the UPA label change is shown in Figure S1.

Study randomization and the start of new TPs were then paused in December 2018 to allow thorough evaluation of the preclinical findings from 2-year rat/mouse carcinogenicity studies with vilaprisan that demonstrated abnormalities of the adrenals, uterus, and skin after lifelong exposure. Although investigations revealed that the preclinical findings were likely to be rodent-specific and of limited clinical relevance, the study sponsor, after discussion with the regulatory authorities, elected to close the study after completion of an additional comprehensive safety follow-up with particular focus on endometrial, adrenal, and skin safety. This was in order to ensure the safety of the women who were treated and to thoroughly assess the potential relevance of the preclinical findings to humans (comprehensive publications on these safety data are in development). The decision to terminate the study early meant that it was not possible to achieve the protocol-planned target sample size for each treatment group and treatment duration for each participant.

During the randomization visit, participants were randomized by the investigator in a 1:1:1:1 ratio to one of the four treatment groups via an interactive voice/web response system. Randomization was stratified by region (i.e., Rest of the World, Taiwan, and South Korea). Block randomization with a block size of eight was used for the initial randomization process, generated by the Sponsor’s Randomization Management group. The study had central randomization, so blocks were shared by patients from the same region. However, with the protocol amendment, further randomization was limited to two groups (i.e., new participants were randomized 1:1 to either the VPR-3/1 or VPR-6/2 group). For this new randomization in the study, a block size of four was used.

The study protocol and its amendments were approved by the Independent Ethics Committee/Institutional Review Board at each participating study site. The study was conducted in accordance with the principles of the Declaration of Helsinki and the International Council for Harmonization Guideline E6: Good Clinical Practice. All patients provided written informed consent to participate in the study.

### 2.2. Study Population

The study enrolled women aged ≥ 18 years with at least one UF of ≥3 cm in diameter as documented by ultrasound at screening. Eligible women had HMB of ≥80 mL, documented by menstrual pictogram (MP). All women were required to be of good general health (except for the UF findings) and have an otherwise normal endometrial histology based on a biopsy performed at screening. Use of a non-hormonal contraceptive method was required starting from Visit 1 until the end of the study. Key exclusion criteria included pregnancy or lactation, UF with a diameter ≥ 12 cm, any undiagnosed abnormal genital bleeding, and concomitant treatment with any hormonal contraceptive or other medications that could have interfered with the conduct of the study or the interpretation of the results (e.g., tranexamic acid, GnRH agonists or anticoagulants).

### 2.3. Study Endpoints

All primary efficacy analyses were based on the MP. The primary efficacy endpoint was the presence of amenorrhea, defined as MBL < 2 mL during the last 28 days of TP1, i.e., during weeks 9–12 of treatment. Secondary efficacy endpoints included the total volume of MBL (normalized to 28 days), mean number of bleeding days (normalized to 28 days), amenorrhea rate (defined as MBL < 2 mL during the last 28 days of each TP) and time to onset of controlled bleeding (defined as MBL < 80 mL from the first day and for all subsequent 28-day periods up to the end of the TP). Other endpoints included percent change in volume of the largest UF compared with baseline as measured by ultrasound, and further endpoints assessed by patient-reported outcomes (PROs) measuring relevant symptoms and aspects of QoL.

### 2.4. Assessments

To monitor treatment compliance, electronic case report form data (i.e., drug accountability) were analyzed and women were required to complete an electronic (e)Diary daily to document study drug intake and symptoms throughout the study. During bleeding episodes, an electronic version of the MP was used by participants to assess the intensity of their MBL per sanitary product, with these data being used to assess the rate of amenorrhea and controlled bleeding. These assessments were reported by day and per bleeding episode. For the primary endpoint analysis, amenorrhea rates were determined during the last 28 days of TP1 for the UPA-3/2 group and for the pooled data from the VPR-3/1, VPR-6/2, and VPR-3/2 groups (hereafter, total vilaprisan-treated group). Data pooling from the vilaprisan groups was possible for the first TP, as differences between regimens occurred only after this timepoint. During TP1, treatment exposure and dosing were identical across vilaprisan regimens; therefore, pooling for analyses based on TP1 was considered appropriate from a clinical and pharmacodynamic perspective. Fibroid volume was assessed by both ultrasound and magnetic resonance imaging (MRI). The three largest fibroids were identified via ultrasound examination in each woman during the screening period; volumetric measurements were subsequently obtained at baseline, during treatment at Visits 4, 5 and 6, and during the end of treatment (EoT) and follow-up visits using a method appropriate to the location of the UF and by the same examiner and device (where possible). The location of the largest fibroid was determined according to International Federation of Gynecology and Obstetrics classification [34]. MRIs were performed at baseline, EoT, and at follow-up visits.

During the study, participants rated their condition using several PRO instruments that were electronically applied. The Uterine Fibroid Daily Bleeding Diary (UF-DBD) and the Uterine Fibroid Daily Symptom Diary (UF-DSD) were completed daily in the eDiary, while the Patient Global Impression of Severity (PGI-S) scale and the Uterine Fibroid Symptom and Quality of Life questionnaire (UFS-QoL) were completed at pre-defined study timepoints, either in the eDiary or on a tablet computer at each study site.

The UF-DBD assesses perceived severity of menstrual bleeding by asking women to rate any vaginal bleeding in the past 24 h on a scale from “no vaginal bleeding” to “very severe” [35]. The number of bleeding days (recorded on the UF-DBD) was counted from Day 1 of TP1 until the day before the start of a new TP.

The UF-DSD is a newly developed multi-item instrument designed to assess cardinal symptoms of UFs using a 24 h recall period. These cardinal symptoms include swelling and bloating (assessed by a five-graded Likert-type severity rating ranging from ‘’no symptom’’ to “very severe symptom’’), and pain at its worst in the abdominal/pelvic regions and the lower back (assessed by a numerical rating scale with 0 indicating “no pain” and 10 “pain as bad as you can imagine”) [36].

PGI-S is a single-item that uses a verbal rating scale to describe the current symptom severity of UFs (options of “none,” “very mild,” “mild,” “moderate,” and “severe”).

The UFS-QoL questionnaire is a widely used disease-specific instrument that assesses symptom severity (SS) and health-related QoL (HRQoL) using a recall period of 3 months. The UFS-QoL comprises 37 items, including an eight-item SS scale and 29 HRQoL questions comprised of six subscales of concern, activities, energy/mood, control, self-consciousness, and sexual function [37]. Individual item scores were added and also transformed into a 0–100-point scale to generate the total SS and HRQoL subscale (raw or transformed) scores; higher SS scores indicate more severe symptoms, and higher HRQoL scores indicate a more favorable QoL.

### 2.5. Safety

Safety parameters were monitored throughout the study. Secondary safety endpoints, including endometrial histology and endometrial thickness, as well as other safety endpoints such as adverse events (AEs), including serious adverse events (SAEs) and treatment-emergent adverse events (TEAEs), are listed in Table S1. The pausing and subsequent closure of the ASTEROID clinical program and the occurrence of the COVID-19 pandemic resulted in a substantially prolonged follow-up phase, often lasting ≥ 1 year. TEAEs were therefore defined as any AE that occurred after the first dose of study drug until 60 days after the last dose, in line with the originally planned follow-up phase of two menstrual cycles. AEs that were first recorded 61 days after the last dose of study drug were defined as post-treatment AEs.

Endometrial safety was monitored throughout the study via the assessment of endometrial histology (single safety read followed by a multi-reader assessment—carried out by three experts—of biopsy specimens collected at screening and during the follow-up period), endometrial thickness (monthly ultrasound investigations), and observation of bleeding patterns. Unscheduled biopsies were performed in women with increased endometrial thickness of >18 mm or a suspicious bleeding pattern (e.g., continuous spotting or unusually heavy bleeding after the start of treatment). Due to the study’s temporary pause and subsequent closure, not all women consented to, or were scheduled for, a post-baseline biopsy. An exit examination time window (from 7 days before final dose until the final study visit) was defined to ensure the safety of all women exposed to the study drug. Women who did not have a normal result for the endometrial biopsy at EoT −7 days or later were required to undergo a repeat biopsy at the study closeout visit.

Three assessment programs were added to the protocol. Monthly monitoring of liver parameters, including a close observation in cases with increased liver enzymes and liver disorders, was introduced following the first PRAC assessment of UPA; this was based on laboratory assessment and a liver symptom questionnaire. In accordance with the procedures outlined in the Food and Drug Administration 2009 drug-induced liver injury guidance [23], close liver observation was necessary if alanine transferase (ALT) or aspartate aminotransferase (AST) values exceeded three times the upper limit of normal (ULN) or increased by two-fold above the lowest baseline level for women with elevated pre-exposure levels. Similarly, observation was required (regardless of ALT or AST levels) if alkaline phosphatase levels exceeded two times the ULN, in cases where alkaline phosphatase levels either were normal at baseline, or increased to at least two times the baseline value where the baseline value was slightly above the ULN. A comprehensive adrenal monitoring program, including adrenal signs and symptoms, adrenal imaging (MRI), and laboratory assessments of adrenal hormones, as well as a thorough skin examination by a dermatologist, were also implemented at the closeout visit based on findings in animal carcinogenicity studies and the sponsor’s decision to close the study.

AEs of special interest (AESIs) related to VPR and UPA included HMB that led to study discontinuation, required intervention (diagnostic procedures or treatment), showed significant clinical worsening during the trial, or was regarded as a SAE; liver disorders that required close observation; adrenal abnormalities assessed as being relevant; endometrial hyperplasia (per World Health Organization 2014 classification [38]); endometrial thickening > 18 mm; and skin disorders including pre-cancerous or malignant skin lesions.

### 2.6. Statistical Analysis

The full analysis set (FAS) was the main dataset used for the primary analysis and for all other efficacy variables; the per-protocol set (PPS) was used for sensitivity analyses, and safety analyses were based on the safety analysis set (SAF). The FAS comprised all randomized women, excluding those who did not start treatment due to the temporary pause. The SAF comprised all randomized women who received ≥ 1 dose of treatment. For safety analyses, data relating to vilaprisan-treated patients were restricted to the time of vilaprisan treatment (i.e., for patients who switched from UPA to vilaprisan, the period of UPA treatment was excluded). The PPS comprised all women in the FAS without any validity findings that impacted the primary efficacy variable. Sample size calculations were based on data for amenorrhea rates from the ASTEROID 2 study (for both vilaprisan and UPA) [30] and were driven by requirements for testing the non-inferiority of vilaprisan with respect to UPA. Assuming an amenorrhea rate of 78.5% in the UPA-3/2 group, a total of 358 women (179 in the VPR-3/2 group and 179 in the UPA-3/2 group) provided an overall power of 95% with a one-sided alpha of 2.5% and non-inferiority margin of −10% when the difference between both amenorrhea rates was at least 5.1% in favor of vilaprisan.

For the primary efficacy analysis, tests were performed in a hierarchical manner. First (step 1), the non-inferiority test was carried out for the primary efficacy variable of amenorrhea in the total vilaprisan-treated group versus UPA-3/2-treated women based on the lower limit of the 95% confidence interval (CI), with a non-inferiority margin of −10%. Second (step 2), the superiority of VPR-3/1-treated women versus UPA-3/2-treated women for the second efficacy variable of total volume of MBL was assessed using analysis of covariance (ANCOVA). The total vilaprisan-treated group was not used for this analysis as differences between vilaprisan treatment regimens, including the number of bleeds during treatment breaks, may have affected this outcome measure. Instead, the VPR-3/1 group was selected for this analysis as this treatment regimen was considered the most likely to be pursued for further development. Finally (step 3), the test for superiority of the primary efficacy variable of amenorrhea was carried out for the total vilaprisan-treated group versus UPA-3/2-treated women using the two-sided Fisher’s exact test with a 0.05 significance level applied. Early study termination and reduced sample size did not affect multiplicity control, as study termination was based on safety considerations and was unrelated to any efficacy assessments. However, the power of the statistical tests was influenced by the actual sample size being smaller than originally planned. Post hoc power calculations for the individual tests based on the observed sample sizes were >99% for the non-inferiority test of the primary efficacy endpoint (amenorrhea rate, step 1), >90% for the superiority test of the key secondary efficacy variable (total volume of MBL, step 2), and 48% for the superiority test of the primary efficacy variable (amenorrhea rate, step 3).

Time-to-event variables were analyzed using Kaplan–Meier estimates. In addition, all efficacy variables were analyzed descriptively, and all statistical analyses were prespecified. Statistical analyses were performed with the SAS release 9.4 software package or higher (SAS Institute Inc., Cary, NC, USA).

## 3. Results

### 3.1. Patient Disposition

**Figure 1:**
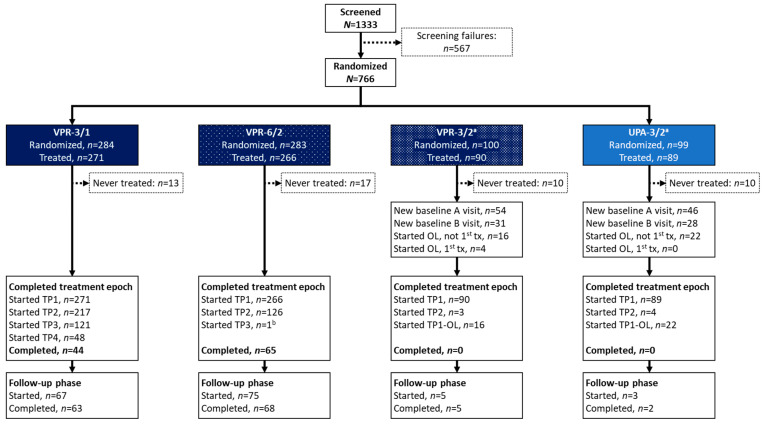
Patient disposition. ^a^ Randomization to the blinded VPR-3/2 and UPA-3/2 groups was stopped due to the suspension of the UPA-3/2 group. A limited number of women randomized to VPR-3/2 or UPA-3/2 treatment started TP2 because UPA treatment was suspended, with women being offered to change to open-label (OL) VPR-3/2 in both groups. In the VPR-3/2 arm, 16 women switched from double-blind to OL VPR-3/2; four additional women had OL VPR-3/2 as their first study drug, but these four were included in the TP1 VPR-3/2 phase and not in the TP1-OL phase. This was decided to enable a homogeneous group of women who received vilaprisan for the first time in TP1, and to avoid combining the first-time treatment starters with those continuing from the double-blind phase into TP1-OL. ^b^ One participant completed the first 168 days in TP1 then mistakenly started taking medication in TP2 after only one bleeding episode. Upon realizing this error, she interrupted TP2 after nine days, waited for another bleeding episode, and then completed 168 days in TP3. OL, open label; TP, treatment period; TP1-OL, received open-label VPR-3/2; tx, treatment; UPA, ulipristal acetate; VPR, vilaprisan.

The trial began in July 2017 and ended on 25 October 2021 following study closure. After informed consent, 1333 women were screened at 87 study centers across 22 countries, and 766 (57.5%) were randomized to one of four treatment groups; patient disposition is shown in Figure 1. The most frequent reasons for study noncompletion were patient withdrawal (*n* = 265, 34.6%), study terminated by sponsor (*n* = 202, 26.4%), and “other” (*n* = 92, 12.0%). A total of 558 women (72.8%) who were randomized to vilaprisan prematurely discontinued the study, most frequently due to participant withdrawal (*n* = 226, 29.5%), study terminated by sponsor (*n* = 184, 24.0%), and “other” (*n* = 77, 10.1%). The FAS included 756 women (98.7% of those randomized); 10 (1.3%) randomized women were excluded from the FAS (did not receive study medication due to the study pause). The PPS included 634 women (82.8%). The most frequent reasons for the exclusion of 132 women (17.2%) from the PPS included major treatment/compliance issues (6.7%), women not receiving any study drug (5.2% never took any study drug and 1.3% did not receive the study drug due to the study pause), and missing efficacy data (3.9%). The SAF included 716 women (93.5%); the 50 women (6.5%) who were randomized but never took the study drug were excluded. Overall mean treatment compliance analyzed using electronic case report form data was 98.6%.

### 3.2. Baseline Demographics and Characteristics

The baseline demographics and disease characteristics of the FAS are provided in Table 1. The baseline mean volume of MBL for the 28-day period measured by MP was 216.2 mL, with 41.1% of all women experiencing MBL of up to 150 mL and 39.6% experiencing MBL of >150 to ≤300 mL. The mean sum volume of the three largest fibroids at baseline (measured by ultrasound) was 121.26 mL and the mean largest fibroid diameter was 41.9 mm. The largest fibroid was located intramurally in 37.7% of women, with subserosal ≥ 50% intramural, subserosal < 50% intramural and submucosal ≥ 50% intramural locations accounting for 23.0%, 13.6%, and 10.6% of women, respectively.

### 3.3. Primary Efficacy Analysis

#### 3.3.1. Test for Non-Inferiority in Amenorrhea Rate in Vilaprisan Versus UPA Treatment Groups

**Figure 2:**
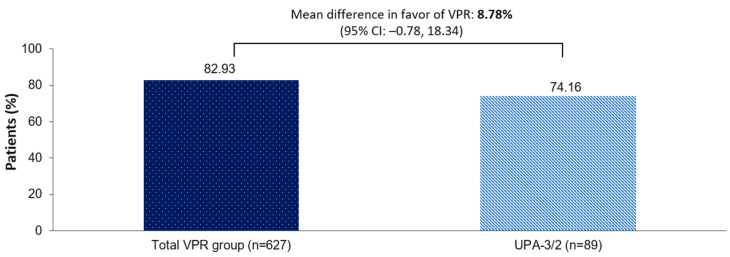
Amenorrhea rate after the first 12 weeks of treatment (step 1 and 3 of the hierarchical test) as assessed by MP. Amenorrhea was assessed by the MP and was defined as bleeding of <2 mL during the last 28 days of TP1 (FAS). The superiority test did not achieve statistical significance in favor of the total VPR-treated group over UPA-3/2 (*p* = 0.0553). However, the 95% CI for the mean difference between the total VPR-treated group and the UPA-3/2 group after 12 weeks of treatment showed that VPR was non-inferior to UPA (non-inferiority threshold: −10%). The total VPR-treated group was defined as all vilaprisan-treated women combined (i.e., VPR-3/1, VPR-6/2, and VPR-3/2). CI, confidence interval; FAS, full analysis set; MP, menstrual pictogram; TP, treatment period; UPA, ulipristal acetate; VPR, vilaprisan.

Amenorrhea as assessed by the MP was reported more frequently in women treated with vilaprisan (total vilaprisan-treated group, *n* = 627) compared with women who received UPA-3/2 (*n* = 89) after the first 12 weeks of treatment, at 82.9% versus 74.2% (rate difference: 8.78%; 95% CI: −0.78%, 18.34%). The lower limit of the 95% CI of the difference between amenorrhea rates (−0.78%) was above the non-inferiority threshold defined for the study (−10%), demonstrating that vilaprisan was non-inferior to UPA for that efficacy variable (Figure 2). As a sensitivity analysis, the rate of amenorrhea in the PPS confirmed that vilaprisan (total vilaprisan-treated group, *n* = 559) was non-inferior to UPA (UPA-3/2, *n* = 75): 85.7% versus 84.0% (rate difference: 1.69%; 95% CI: −7.10%, 10.48%).

#### 3.3.2. Test for Superiority in Total Volume of MBL in Vilaprisan Versus UPA Treatment Groups

**Figure 3:**
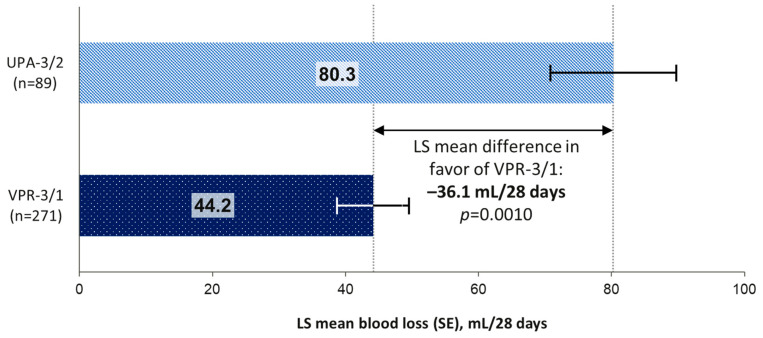
Total volume of menstrual blood loss (normalized to 28 days) for TP1 (step 2 of the hierarchical test). Total volume of MBL was based on the MP. LS, least squares; MBL, menstrual blood loss; MP, menstrual pictogram; TP, treatment period; UPA, ulipristal acetate; VPR, vilaprisan.

Superiority testing of vilaprisan versus UPA for the total volume of MBL as assessed by the MP was performed (note that only the VPR = 3/1 arm was included in this analysis [please see Section 2 for details]). The least squares mean (standard error) total MBL (normalized to 28 days) for TP1 was significantly lower in the VPR-3/1 group compared with the UPA-3/2 group (44.2 [5.4] mL versus 80.3 [9.5] mL; difference −36.1 mL; *p* = 0.0010; Figure 3). Sensitivity analysis in the PPS also significantly favored the VPR-3/1 group compared with the UPA-3/2 group (least squares mean difference −19.9 mL; *p* ≤ 0.0001).

#### 3.3.3. Test for Superiority in Amenorrhea Rate in Vilaprisan Versus UPA Treatment Groups

Finally, in step 3 of the hierarchical testing procedure, superiority for the primary variable of amenorrhea was assessed for the total vilaprisan-treated group versus the UPA-3/2 group. No statistically significant difference was observed in the amenorrhea rate between groups using either the FAS (*p* = 0.0553) or PPS (*p* = 0.7268) datasets.

### 3.4. Secondary Endpoint Analyses

The mean ± standard deviation (SD) total volume of MBL (normalized to 28 days), as assessed by the MP, was highest in the UPA-3/2 group, at 80.5 ± 157.7 mL, followed by the VPR-3/2 group (same treatment/bleeding episode schedule as for the UPA-3/2 group) at 65.9 ± 89.0 mL, and lowest in the VPR-3/1 and VPR-6/2 groups, at 41.2 ± 62.4 and 46.3 ± 54.1 mL, respectively. The mean ± SD number of bleeding days (normalized to 28 days), as assessed by the UF-DBD, was 2.4 ± 2.1 days in the UPA-3/2 group and 2.3 ± 3.0 days in the VPR-3/2 group. In the other vilaprisan-treatment groups, the values were as follows: total vilaprisan-treated group, 1.8 ± 1.9 days; VPR-3/1, 1.6 ± 1.5 days; and VPR-6/2, 1.8 ± 1.8 days.

**Figure 4:**
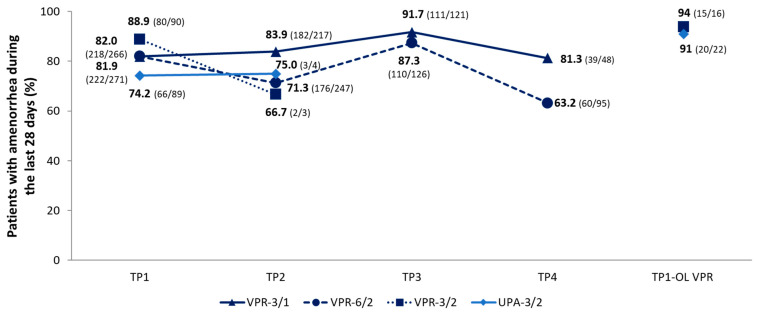
Number and proportion of patients with amenorrhea during the last 28 days (based on the MP) by treatment group and treatment period. Patients in the VPR-3/2 and UPA-3/2 groups all received OL VPR-3/2 during the TP1-OL VPR phase. The 95% confidence intervals for these data are provided in Table S2. MP, menstrual pictogram; OL, open label; TP, treatment period; UPA, ulipristal acetate; VPR, vilaprisan.

During TP1, amenorrhea rates were 81.9% in the VPR-3/1 group, 88.9% in the VPR-3/2 group, and 82.0% in the VPR-6/2 group, compared with 74.2% in the UPA-3/2 group. The highest and lowest amenorrhea rates, respectively, occurred during TP3 (91.7%) and TP4 (81.3%) for the VPR-3/1 group; TP3 (87.3%) and TP4 (63.2%) for the VPR-6/2 group; TP1-OL (93.8%) and TP2 (66.7%) for the VPR-3/2 group; and TP1-OL (90.9%) and TP1 (74.2%) for the UPA-3/2 group (Figure 4). The median time to onset of controlled bleeding in TP1 was 1 day for vilaprisan-treated women (regardless of the treatment regimen) and women receiving UPA-3/2. In TP2, the median time to onset of controlled bleeding was 1 day and 1.5 days for women receiving vilaprisan and UPA-3/2, respectively.

### 3.5. Other Endpoints

#### 3.5.1. Percentage Change in Volume of the Largest Fibroid

**Figure 5:**
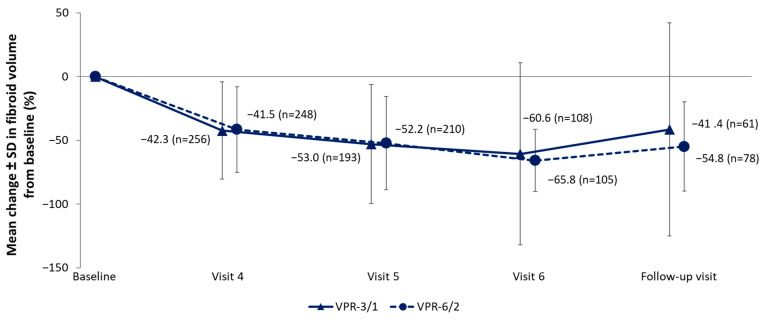
Percent change in volume of the largest fibroid from baseline (measured by ultrasound). Data for the VPR-3/2 and UPA-3/2 groups are not shown here due to the limited sample size and shorter treatment duration; these data are instead illustrated in Figure S2. Visit 4: Week 13 after the start of TP1; Visit 5: Week 13 after the start of TP2 for the VPR-3/1 group and Week 25 after the start of TP1 for the VPR-6/2 group; Visit 6: Week 13 after the start of TP3 for the VPR-3/1 group and Week 13 after the start of TP2 for the VPR-6/2 group. The follow-up visit was conducted between Week 10 to 14 after the EoT. EoT, end of treatment; SD, standard deviation; TP, treatment period; UPA, ulipristal acetate; VPR, vilaprisan.

The volume of the largest fibroid (measured by ultrasound) showed a substantial reduction from baseline at Visit 4 for all groups (mean reduction: −42.3%, −41.5%, −45.8%, and −39.7% for the VPR-3/1, VPR-6/2, VPR-3/2, and UPA-3/2 groups, respectively). For the VPR-3/1 and VPR-6/2 groups, further reductions were observed at Visit 6 (−60.6% for VPR-3/1 and −65.8% for VPR-6/2), with a return to Visit 4 levels at the follow-up visit (−41.4% for VPR-3/1 and −54.8% for VPR-6/2) (Figure 5). Measurement of the largest fibroid volume by MRI showed similar trends with a lower percentage reduction compared with ultrasound.

#### 3.5.2. Changes in UFS-QoL Ratings

Numerical reductions in mean raw and transformed UFS-QoL SS scores relative to baseline, indicating symptom improvement, were observed across the TPs and break periods for all four treatment groups. In treatment groups VPR-3/1, VPR-6/2, VPR-3/2, and UPA-3/2, transformed UFS-QoL SS scores at baseline were 49.7, 51.5, 54.2, and 61.0, respectively, and these scores numerically reduced to 18.3, 18.1, 19.1, and 21.4, respectively, during the third 28-day period of TP1. Values were similar for subsequent TPs and slightly higher during break periods. During the first follow-up period, UFS-QoL SS scores increased to 27.3 and 38.8 for the VPR-3/1 and VPR-6/2 groups, respectively (data for the VPR-3/2 and UPA-3/2 groups are not reported due to there being fewer than 10 women in each group at follow-up). Transformed UFS-QoL HRQoL scores followed a similar trend, with numerical increases observed from baseline, indicating symptom improvement in all treatment groups during the treatment and break periods. For the UFS-QoL concern subscale specifically (baseline scores: 45.2, 41.9, 41.0, and 32.5 for women in the VPR-3/1, VPR-6/2, VPR-3/2, and UPA-3/2 treatment groups, respectively), these scores numerically increased to 82.7, 87.6, 85.2, and 80.2, respectively, during the third 28-day period of TP1. During the first follow-up period, concern subscale scores decreased to 67.8 and 61.5 for the VPR-3/1 and VPR-6/2 groups, respectively (data for the VPR-3/2 and UPA-3/2 groups are not reported due to there being fewer than 10 women in each group at follow-up).

#### 3.5.3. Changes in UF-DSD Ratings

Across treatment groups at baseline, moderate swelling or bloating was reported by 10–20% of women, with <10% of women reporting severe swelling or bloating. Modest mean numerical improvements in UF-DSD scores were noted during treatment for swelling, bloating and pain in the abdominal/pelvic area and lower back compared with baseline. The extent of numerical improvement was similar in the VPR-3/1 and VPR-6/2 groups, with mean values generally returning to baseline levels at the start of the follow-up period. Compared with baseline, a modest mean numerical reduction in the intake of pain medication during treatment was also reported.

#### 3.5.4. Changes in PGI-S Ratings

For the PGI-S ratings, fewer women in the VPR-3/1 group reported severe or very severe symptoms compared with the other treatment groups at baseline (Table S3). Throughout the TPs, all groups experienced a numerical decrease in the number of women with severe or very severe symptoms and an increase in the number of those participants with no or very mild symptoms. When comparing TP2 to TP1, particularly within the VPR-3/1 and VPR-6/2 groups, there was a reduction in the number of women with severe or very severe symptoms, whereas the proportion of women with none or very mild symptoms increased.

### 3.6. Safety Outcomes

#### 3.6.1. Treatment Exposure

The mean ± SD overall extent of exposure for vilaprisan in 552 women was 180.6 ± 89.5 tablets (median 168.0 tablets; range 1–338 tablets). The mean ± SD overall extent of exposure for UPA-3/2 in 83 women was 96.3 ± 47.3 tablets (median 84.0 tablets; range 3–252 tablets). The number of subjects with exposure information is smaller than the number included in the SAF set, as several blisters could not be clearly assigned to a specific treatment period.

#### 3.6.2. TEAEs

A summary of safety data (TEAEs) is provided in Table 2. Overall, TEAEs were reported for 427 (65.8%) women in the total vilaprisan-treated group and for 48 (53.9%) women treated with UPA-3/2. TEAEs were reported more frequently in groups with a longer treatment duration (i.e., VPR-3/1: 70.1%; VPR-6/2: 69.2%) compared with those with a shorter treatment duration (open-label groups for VPR-3/2: 47.4% and UPA-3/2: 39.1%). TEAEs related to ovarian cysts were reported for 11 (1.7%) women in the total vilaprisan-treated group and for one (1.2%) woman treated with UPA-3/2. Serious TEAEs occurred in 26 (4.0%) women in the total vilaprisan-treated group and no such events were reported in UPA-3/2-treated women; no serious TEAE related to ovarian cysts occurred. Serious study drug-related TEAEs were reported for three (0.5%) women in the total vilaprisan-treated group and no such events were reported in UPA-3/2-treated women. All serious study drug-related TEAEs occurred in the system organ class of reproductive system and breast disorders. The preferred terms for these events included abnormal uterine bleeding (reported by one woman [0.4%] in the VPR-3/1 group), uterine hemorrhage (reported by one woman [0.4%] in the VPR-6/2 group) and endometrial hyperplasia without atypia (reported by one woman [0.4%] in the VPR-3/1 group). No deaths occurred during the study.

Overall, 41 women in the total vilaprisan-treated group (6.3%) and five women in the UPA-3/2-treated group (5.6%) discontinued the study drug due to TEAEs (Table 2). The most frequent TEAEs leading to discontinuation of the study drug were hot flushes (vilaprisan, *n* = 10; UPA, *n* = 0), headache (vilaprisan, *n* = 5; UPA, *n* = 3) and nausea (vilaprisan, *n* = 3; UPA, *n* = 0). Three women in the vilaprisan-treated group (0.5%) discontinued the study drug due to serious TEAEs.

#### 3.6.3. AESIs

AESIs occurred in 216 (33.3%) of the total vilaprisan-treated group and in 27 (30.3%) women treated with UPA-3/2, whereas treatment-emergent AESIs occurred in 87 (13.4%) women in the total vilaprisan-treated group and 7 (7.9%) in the UPA-3/2 group.

- HMB

HMB-related treatment-emergent AESIs were reported for 49 (7.6%) women in the total vilaprisan-treated group and for two (2.2%) women treated with UPA-3/2. Of the 49 women in the total vilaprisan-treated group, 25 (3.9%) women had mild, 18 (2.8%) had moderate, and 6 (0.9%) had severe AESIs, while both events in women treated with UPA-3/2 were of mild intensity.

- Liver parameters and disorders

Liver enzyme-related treatment-emergent AESIs were reported for 34 (5.2%) women in the total vilaprisan-treated group and one (1.1%) woman treated with UPA-3/2. Of the 34 women in the total vilaprisan-treated group, 25 (3.9%) women had mild, 8 (1.2%) had moderate, and one (0.2%) had severe AESIs, while the event in the woman treated with UPA-3/2 was of mild intensity. For most women, liver enzyme and bilirubin values were below or equal to the ULN. Increases in liver enzymes or bilirubin were infrequent with no clear trends among the vilaprisan-treated groups.

- Endometrial disorders

Throughout the study, adequate endometrial tissue samples were obtained for the safety read from all 627 women in the total vilaprisan-treated group and 89 women in the UPA-3/2 group. Based on the World Health Organization 2014 criteria [38], there were no cases of hyperplasia with atypia or of malignant neoplasm diagnosed, either in the safety read or majority read. Endometrial hyperplasia without atypia was reported as a treatment-emergent AESI for three (0.5%) women in the total vilaprisan-treated group. Endometrial thickening > 18 mm occurred in 58 (8.9%) women in the total vilaprisan-treated group and in eight (9.0%) women treated with UPA-3/2. Mean ± SD baseline values for endometrial thickness were 10.7 ± 3.2 mm in the total vilaprisan-treated group and 10.4 ± 3.5 mm in the UPA-3/2 group. These mean values decreased during TP1 in all treatment groups, and then returned to baseline levels in the follow-up phase (total vilaprisan group: 11.0 ± 4.8 mm; UPA-3/2: 11.9 ± SD 5.7 mm). At the exit examination, the mean ± SD change from baseline in endometrial thickness was −0.6 ± 4.5 mm in the total vilaprisan-treated group (*n* = 610) and −0.4 ± 5.3 mm in the UPA-3/2 group (*n* = 88). At baseline, 1.8% of women in the total vilaprisan-treated group (equally distributed between vilaprisan treatment groups) and 3.4% in the UPA-3/2 group had endometrial thickness measurements > 18 mm. During the follow-up phase, these percentages increased to 6.9% in the total vilaprisan-treated group and 10.5% in the UPA-3/2 group. At the exit examination, 1.8% of women in the total vilaprisan-treated group and 2.3% in the UPA-3/2 group had endometrial thickness measurements >18 mm.

- Progesterone receptor modulator-associated endometrial changes (PAEC)

Throughout the study, PAEC, which were investigated systematically, were reported for 89 of 617 (14.4%) total vilaprisan-treated women and five of 87 (5.7%) UPA-3/2-treated women. At baseline, PAEC were reported in four of 590 (0.7%) total vilaprisan-treated women assessed and none of the 89 UPA-treated women. During the treatment phase, PAEC were reported in 38 of the 49 (77.6%) total vilaprisan-treated women who had an endometrial biopsy collected and sufficient tissue available for assessment. During the exit examination window, PAEC were reported for 21 of 499 (4.2%) total vilaprisan-treated women and one of 65 (1.5%) UPA-treated women.

- Adrenal disorders

Of the 443 women in the total vilaprisan-treated group and the 63 UPA-treated women who consented to the safety follow-up, 23 and two women, respectively, were diagnosed with an adrenal disorder. No cases of malignant adrenal tumors were reported and no signals for adrenal safety were identified from the study.

- Skin disorders

Of the 506 women who consented to the safety closeout visit, a skin assessment was performed for 491 women. No cases of cutaneous or subcutaneous sarcoma were identified.

- Pregnancies

Two pregnancies were reported during the study. A woman receiving VPR-6/2 tested positive for pregnancy during the temporary study pause, two months after the last dose of study drug. This participant had completed the first treatment period of 24 weeks but did not complete treatment as the study was put on hold. The woman gave birth to a healthy male baby at gestational age 40 weeks via a vaginal delivery. Another woman (in the UPA-3/2 group) tested positive for pregnancy 9 months after the EoT. The participant had a spontaneous abortion a month later. The event was classified as moderate in intensity and was assessed as unrelated to the study drug.

## 4. Discussion

The ASTEROID 5 study examined three different regimens of vilaprisan, an investigational SPRM, for the treatment of UFs in comparison to the approved regimen of UPA. In this study, the total VPR group was non-inferior to UPA-3/2 with respect to amenorrhea rates, with a difference between treatment groups of 8.8% favoring vilaprisan. In addition, the VPR-3/1 regimen (the only regimen tested for superiority here) demonstrated statistically significant superiority over UPA-3/2 in terms of reducing the volume of MBL, with a difference of −36.1 mL/28 days (*p* = 0.001). The other vilaprisan regimens showed numerically favorable outcomes for bleeding-related endpoints; however, these findings were descriptive and not powered to demonstrate superiority. From a clinical perspective, a reduction of 36.1 mL in MBL over a 28-day period corresponds to nearly half of the conventional diagnostic threshold for HMB (>80 mL per cycle). Such a reduction is, therefore, likely to be clinically meaningful, particularly for women close to this threshold and those experiencing bleeding-related symptoms [39]. Reductions in menstrual blood loss have previously been associated with improvements in hemoglobin levels, rates of anemia, and health-related quality of life in women with heavy menstrual bleeding [40,41].

These findings corroborate those of the earlier phase 2b ASTEROID 2 trial, in which HMB response (predefined as MBL < 80 mL and >50% reduction from baseline during the last 28 days of treatment) was achieved by 95.7% of women treated with vilaprisan, compared with 86.5% of those treated with UPA [30]. These outcomes also align with observations from the phase 3 ASTEROID 3 trial, where a notably higher proportion of women treated with vilaprisan achieved a response in HMB compared with those who received placebo (91.7% versus 25.0%, *p* < 0.0001) [29]. Furthermore, in the ASTEROID 4 trial, women receiving vilaprisan showed a significantly higher rate of HMB response compared with those who received placebo (88.9% versus 21.2%, *p* < 0.0001); vilaprisan also induced amenorrhea more frequently compared with placebo (85.7% versus 3.0%, *p* < 0.0001). These collective results highlight vilaprisan as an effective treatment for HMB associated with UFs.

Additionally, a strong treatment effect in the reduction in fibroid volume was observed in ASTEROID 5 in the VPR-3/1 and VPR-6/2 groups. This outcome is consistent with findings from previous studies [27,30,31,32] and suggests that vilaprisan improves UF-related symptoms while addressing the underlying disease.

UFS-QoL SS scores during treatment numerically decreased from baseline in all groups to within a range that can be considered normal [42]. Similar trends were observed for the UFS-QoL HRQoL scores. Although a formally established minimal clinically important difference (MCID) for the UFS-QoL has not been defined, previous analyses have suggested that a 10-point change may represent a reasonable estimate of the MCID [43]. The changes observed in ASTEROID 5 exceeded this threshold, suggesting that they are likely to be clinically meaningful. In Phase 3 clinical trials of GnRH antagonists for UFs, reductions in MBL were similarly accompanied by improvements in symptom severity and HRQoL measures [13,29].

Small, transient numerical improvements from baseline in UF-DSD scores were reported during study treatment in the VPR-3/1 and VPR-6/2 groups, with mean values generally returning to baseline levels during follow-up, indicating treatment response with regard to symptom severity. A modest mean numerical reduction in the intake of pain medication during treatment was also noted. Overall, a similar pattern for all PRO measures was seen, supporting the positive results from the primary and secondary endpoint analyses.

Predominantly mild to moderate AEs were observed with vilaprisan treatment, with minimal impact observed on liver enzymes. Although a small percentage of women in the total vilaprisan and UPA-3/2 groups (5.2% and 3.2%, respectively) showed abnormal adrenal imaging features, most cases were resolved upon expert review, and rates were in line with the expected 1–6% prevalence of incidentally discovered adrenal masses in the general population [44]. In the majority of cases with AESIs, women had recovered or were recovering from the AESIs before the end of the study. Importantly, it must be noted that the generalizability of these safety findings is limited by the reduced sample sizes and early study termination, and the absence of observed safety signals should not be interpreted as evidence of absence of risk. Furthermore, the prolonged follow-up period after treatment discontinuation may have led to the capture of post-treatment AEs unrelated to treatment, which could have influenced the overall incidence of reported AEs and thereby hampered the generalizability of the safety data.

The study’s strengths include its multicenter, randomized, partially double-blind, active-controlled design, which allowed for a comparison of three different vilaprisan regimens with UPA in a multi-country population. The study utilized robust efficacy endpoints, including amenorrhea and HMB response, which were clearly defined prior to the study and assessed using the MP and the UF-DBD. These endpoints were additionally supported by improvements in PROs, including those measuring symptom severity and HRQoL. Furthermore, the study featured extensive safety monitoring, ensuring a comprehensive assessment of the safety profiles of the treatments. The sample size reached in December 2018, in combination with the prespecified hierarchical testing strategy, permitted testing of the primary study objective as planned (statistical power of >95% for the non-inferiority test). In addition, the available data allowed descriptive characterization of fibroid symptoms with the VPR-3/2 regimen. However, further research would be needed to understand the long-term safety and efficacy of these regimens, as well as the optimal duration and frequency of treatment cycles. Considering variability in patient response, the availability of personalized treatment choices is key to achieving optimal outcomes versus the alternative VPR-3/1 and VPR-6/2 options.

This study has several limitations. The number of women in the study was lower than planned due to the temporary study pause and ultimate termination of the trial, which prevented further enrollment. Consequently, the maximum treatment duration in the study was approximately 1 year, although 2 years were originally planned. Although the sample sizes remained sufficiently large to compare safety aspects of the VPR 3/1 and VPR-6/2 regimens, a meaningful comparison with the UPA 3/2 regimen is limited by the smaller sample size in the latter arm. Restrictions on the use of UPA due to a regulatory change in the product label necessitated early cessation of enrollment to the UPA-3/2 group, resulting in a lower comparator sample size than originally planned. However, the statistical power for the primary non-inferiority analysis was maintained, and the superiority testing could still be conducted within the prespecified hierarchical framework. In addition, an imbalance in the location of the largest UF was observed across treatment arms at baseline, with a higher proportion of women in the UPA-3/2 group having a largest UF contacting the endometrium with a 100% intramural location. Although baseline MBL values were comparable across treatment groups, fibroids in close proximity to the endometrium have been associated with heavier bleeding and may influence MBL [4,45]. Therefore, this imbalance may have contributed to variability in bleeding outcomes and should be considered when interpreting the comparative efficacy results. Differences in treatment exposure duration between regimens may have also influenced the observed efficacy outcomes. For example, the degree of natural disease progression may have been different between the groups with longer versus shorter treatment exposure, and it is difficult to predict the direction of this potential effect. This should be considered when interpreting the comparative efficacy results. Finally, it is important to note that the ultrasound assessments yielded variable results, and many values had notably high SDs; MRI assessments may have offered a more reliable method of evaluating treatment outcomes; however, MRI assessments were limited in the VPR-3/2 group, with only 13 patients assessed at the EoT visit and one at the closeout visit. Overall, these factors limit the generalizability of the efficacy and safety findings reported here.

Recent reports have described the use of diverse medical strategies for uterine fibroids, including progesterone receptor antagonists/modulators, GnRH agonists, and antagonists, and combination regimens integrating pharmacological therapy with interventional procedures such as uterine artery embolization [46,47] Although mechanistically distinct from SPRMs, these therapies reflect the evolving medical treatment landscape for uterine fibroids and underscore the ongoing need for effective and well-tolerated pharmacological options.

The ASTEROID 5 study is the first to compare SPRM regimens and may provide exploratory insights into how the treatment schedule and duration may influence clinical outcomes. Despite the termination of the vilaprisan development program following findings from long-term rodent carcinogenicity studies, the efficacy results observed in ASTEROID 5 remain clinically informative. To our knowledge, no other SPRM is currently in advanced clinical development for uterine fibroids, which is unfortunate given the positive efficacy findings in this study, including clinically meaningful reductions in menstrual blood loss and substantial reductions in fibroid volume. Although vilaprisan development was discontinued as a precautionary measure, these data reinforce the relevance of SPRMs as a therapeutic target for the treatment of uterine fibroids. Other emerging medical strategies, including GnRH antagonists and combination approaches, are discussed above; however, there remains a significant unmet need for effective, well-tolerated treatments that enhance the overall quality of life of women with UFs.

## Figures and Tables

**Table 1:** Demographics and baseline characteristics for the FAS.

	Treatment Group					
Characteristic	VPR-3/1 *n* = 280	VPR-6/2 *n* = 279	VPR-3/2 *n* = 99	UPA-3/2 *n* = 98	Total VPR-Treated ^a^ *N* = 658	Total *N* = 756
Age (years)						
Mean ± SD	43.2 ± 5.3	43.0 ± 5.7	43.7 ± 5.4	43.6 ± 5.0	43.2 ± 5.5	43.2 ± 5.4
Range	24–53	20–60	23–53	26–53	20–60	20–60
Race, *n* (%)						
White	202 (72.1)	199 (71.3)	93 (93.9)	88 (89.8)	494 (75.1)	582 (77.0)
Black or African American	11 (3.9)	11 (3.9)	1 (1.0)	4 (4.1)	23 (3.5)	27 (3.6)
Asian	65 (23.2)	64 (22.9)	5 (5.1)	6 (6.1)	134 (20.4)	140 (18.5)
American Indian or Alaska Native	1 (0.4)	0	0	0	1 (0.2)	1 (0.1)
Not reported	1 (0.4)	3 (1.1)	0	0	4 (0.6)	4 (0.5)
Multiple	0	2 (0.7)	0	0	2 (0.3)	2 (0.3)
Weight at baseline (kg)						
Mean ± SD	69.8 ± 14.0	69.3 ± 14.4	71.3 ± 15.0	71.7 ± 13.6	69.8 ± 14.3	70.1 ± 14.3
Range	41.7–127.0	38.8–123.0	48.0–118.0	48.0–105.5	38.8–127.0	38.8–127.0
Baseline BMI (kg/m^2^)						
Mean ± SD	25.6 ± 4.7	25.7 ± 4.9	25.9 ± 4.9	26.4 ± 5.0	25.7 ± 4.8	25.8 ± 4.9
Range	17.0–48.4	16.8–45.5	19.2–42.8	17.0–39.4	16.8–48.4	16.8–48.4
Baseline MBL for 28 days by MP (mL), *n*	278	275	99	97	652	749
Mean ± SD	212.8 ± 152.0	214.4 ± 141.8	218.9 ± 130.7	228.6 ± 142.3	214.4 ± 144.4	216.2 ± 144.1
Range	45.7–935.5	11.3–916.0	3.8–611.5	77.1–919.7	3.8–935.5	3.8–935.5
Categorized baseline MBL for 28 days by MP (mL), *n* (%)						
Missing	2 (0.7)	4 (1.4)	0	1 (1.0)	6 (0.9)	7 (0.9)
≤150	131 (46.8)	109 (39.1)	34 (34.3)	37 (37.8)	274 (41.6)	311 (41.1)
>150 to 300	100 (35.7)	118 (42.3)	46 (46.5)	35 (35.7)	264 (40.1)	299 (39.6)
>300 to 500	26 (9.3)	35 (12.5)	12 (12.1)	21 (21.4)	73 (11.1)	94 (12.4)
>500	21 (7.5)	13 (4.7)	7 (7.1)	4 (4.1)	41 (6.2)	45 (6.0)
Largest fibroid diameter by ultrasound (mm)						
Mean ± SD	40.9 ± 20.4	44.7 ± 21.7	39.3 ± 20.0	39.2 ± 22.7	42.3 ± 21.0	41.9 ± 21.2
Range	0–107	9–112	14–109	0–116	0–112	0–116
Location of largest fibroid, *n* (%)						
Intramural	103 (36.8)	115 (41.2)	37 (37.4)	30 (30.6)	255 (38.8)	285 (37.7)
Subserous ≥50% intramural	70 (25.0)	59 (21.1)	21 (21.2)	24 (24.5)	150 (22.8)	174 (23.0)
Subserous <50% intramural	40 (14.3)	38 (13.6)	13 (13.1)	12 (12.2)	91 (13.8)	103 (13.6)
Submucous ≥50% intramural	34 (12.1)	24 (8.6)	15 (15.2)	7 (7.1)	73 (11.1)	80 (10.6)
Contacts endometrium, 100% intramural	22 (7.9)	25 (9.0)	6 (6.1)	19 (19.4)	53 (8.1)	72 (9.5)
Other ^b^	11 (3.9)	18 (6.5)	7 (7.1)	5 (5.1)	36 (5.5)	41 (5.4)
Missing	0	0	0	1 (1.0)	0	1 (0.1)
Baseline hemoglobin (g/dL)						
Mean ± SD	12.2 ± 1.7	11.9 ± 1.9	12.3 ± 1.7	12.5 ± 1.6	12.1 ± 1.8	12.1 ± 1.8
Range	6.8–15.1	5.8–15.7	7.6–15.3	7.5–16.2	5.8–15.7	5.8–16.2

^a^ Total VPR-treated women combined, regardless of treatment and bleeding episode regimen (i.e., VPR-3/1 + VPR-6/2 + VPR-3/2). ^b^ Other locations across the entire study population included submucous <50% intramural (*n* = 14, 1.9%), subserous pedunculated (*n* = 14, 1.9%), submucous pedunculated intracavitary (*n* = 5, 0.7%), hybrid myoma/transmural myoma (*n* = 5, 0.7%), other cervical (*n* = 2, 0.3%), and other parasitic (*n* = 1, 0.1%). BMI, body mass index; FAS, full analysis set; MBL, menstrual blood loss; MP, menstrual pictogram; SD, standard deviation; UPA, ulipristal acetate; VPR, vilaprisan.

**Table 2:** Summary of treatment-emergent adverse events.

Data Are *n* (%)	Treatment Group						
Type of AE	VPR-3/1 *n* = 271	VPR-6/2 *n* = 266	VPR-3/2 (DB) *n* = 86	UPA-3/2 (DB) *n* = 89	VPR-3/2 (OL) *n* = 19	VPR-3/2 (B-OL) *n* = 23	Total VPR-Treated ^a^ *N* = 649
Any TEAE	190 (70.1)	184 (69.2)	39 (45.3)	48 (53.9)	9 (47.4)	9 (39.1)	427 (65.8)
Any TEAE leading to study drug discontinuation	21 (7.7)	17 (6.4)	3 (3.5)	5 (5.6)	0	0	41 (6.3)
Most frequent TEAEs (≥1% in any group) according to preferred term (MedDRA version 24.0)							
Anemia	3 (1.1)	5 (1.9)	3 (3.5)	0	0	0	11 (1.7)
Abdominal pain lower	8 (3.0)	5 (1.9)	1 (1.2)	1 (1.1)	1 (5.3)	0	15 (2.3)
Abdominal pain	6 (2.2)	9 (3.4)	3 (3.5)	2 (2.2)	0	0	18 (2.8)
Diarrhea	4 (1.5)	2 (0.8)	0	1 (1.1)	1 (5.3)	0	7 (1.1)
Abdominal discomfort	2 (0.7)	1 (0.4)	0	1 (1.1)	1 (5.3)	0	4 (0.6)
Vomiting	2 (0.7)	1 (0.4)	0	0	1 (5.3)	1 (4.3)	5 (0.8)
Fatigue	14 (5.2)	17 (6.4)	0	0	0	0	31 (4.8)
Pyrexia	0	5 (1.9)	0	0	1 (5.3)	0	6 (0.9)
Nasopharyngitis	16 (5.9)	15 (5.6)	2 (2.3)	4 (4.5)	2 (10.5)	0	35 (5.4)
Cystitis	1 (0.4)	0	1 (1.2)	0	0	1 (4.3)	3 (0.5)
Gastroenteritis viral	1 (0.4)	0	0	0	0	1 (4.3)	2 (0.3)
Alanine aminotransferase increased	5 (1.8)	9 (3.4)	2 (2.3)	0	1 (5.3)	0	17 (2.6)
Blood thyroid stimulating hormone increased	5 (1.8)	5 (1.9)	0	0	0	1 (4.3)	11 (1.7)
Blood cholesterol increased	0	0	0	0	1 (5.3)	0	1 (0.2)
Hypertriglyceridemia	1 (0.4)	2 (0.8)	1 (1.2)	0	1 (5.3)	0	5 (0.8)
Type 2 diabetes mellitus	0	1 (0.4)	0	0	0	1 (4.3)	2 (0.3)
Temporomandibular joint syndrome	0	1 (0.4)	0	0	0	1 (4.3)	2 (0.3)
Headache	34 (12.5)	43 (16.2)	11 (12.8)	11 (12.4)	1 (5.3)	2 (8.7)	91 (14.0)
Pollakiuria	3 (1.1)	0	0	0	1 (5.3)	0	4 (0.6)
Endometrial thickening	18 (6.6)	33 (12.4)	3 (3.5)	6 (6.7)	0	0	54 (8.3)
Dysmenorrhea	4 (1.5)	1 (0.4)	0	3 (3.4)	0	0	5 (0.8)
Uterine hemorrhage	4 (1.5)	6 (2.3)	0	1 (1.1)	0	1 (4.3)	11 (1.7)
Amenorrhea	1 (0.4)	1 (0.4)	0	0	1 (5.3)	0	3 (0.5)
Fibrocystic breast disease	1 (0.4)	0	0	0	0	1 (4.3)	2 (0.3)
Hemorrhagic ovarian cyst	0	0	0	0	0	1 (4.3)	1 (0.2)
Hysterosalpingectomy	2 (0.7)	0	0	0	0	1 (4.3)	3 (0.5)
Hot flush	32 (11.8)	46 (17.3)	5 (5.8)	3 (3.4)	1 (5.3)	2 (8.7)	85 (13.1)
Any serious TEAE	15 (5.5)	10 (3.8)	0	0	0	1 (4.3)	26 (4.0)
Serious TEAEs according to preferred term							
Autoimmune thyroiditis	1 (0.4)	0	0	0	0	0	1 (0.2)
Ileus	0	1 (0.4)	0	0	0	0	1 (0.2)
Cholecystitis	0	1 (0.4)	0	0	0	0	1 (0.2)
Hepatic steatosis	0	1 (0.4)	0	0	0	0	1 (0.2)
Cellulitis	0	1 (0.4)	0	0	0	0	1 (0.2)
Gastroenteritis	0	1 (0.4)	0	0	0	0	1 (0.2)
Ankle fracture	1 (0.4)	0	0	0	0	0	1 (0.2)
Upper limb fracture	1 (0.4)	0	0	0	0	0	1 (0.2)
ASAT increased	0	1 (0.4)	0	0	0	0	1 (0.2)
Ovarian germ cell teratoma benign	1 (0.4)	0	0	0	0	0	1 (0.2)
Basal cell carcinoma	0	1 (0.4)	0	0	0	0	1 (0.2)
Vertigo CNS origin	0	1 (0.4)	0	0	0	0	1 (0.2)
Suicidal ideation	0	1 (0.4)	0	0	0	0	1 (0.2)
Stress urinary incontinence	1 (0.4)	0	0	0	0	0	1 (0.2)
Ureterolithiasis	1 (0.4)	0	0	0	0	0	1 (0.2)
Abnormal uterine bleeding	2 (0.7)	0	0	0	0	0	2 (0.3)
Heavy menstrual bleeding	2 (0.7)	0	0	0	0	0	2 (0.3)
Endometrial hyperplasia	1 (0.4)	0	0	0	0	0	1 (0.2)
Uterine hemorrhage	0	1 (0.4)	0	0	0	0	1 (0.2)
Uterine polyp	0	1 (0.4)	0	0	0	0	1 (0.2)
Hysterectomy	2 (0.7)	1 (0.4)	0	0	0	0	3 (0.5)
Hysterosalpingectomy	2 (0.7)	0	0	0	0	1 (4.3)	3 (0.5)
Any serious TEAE leading to study drug discontinuation	1 (0.4)	2 (0.8)	0	0	0	0	3 (0.5)
Any study-drug related TEAE	91 (33.6)	105 (39.5)	16 (18.6)	29 (32.6)	3 (15.8)	4 (17.4)	217 (33.4)
Any study-drug related serious TEAE	2 (0.7)	1 (0.4)	0	0	0	0	3 (0.5)
Study-drug related serious TEAEs according to preferred term							
Abnormal uterine bleeding	1 (0.4)	0	0	0	0	0	1 (0.2)
Endometrial hyperplasia	1 (0.4)	0	0	0	0	0	1 (0.2)
Uterine hemorrhage	0	1 (0.4)	0	0	0	0	1 (0.2)

^a^ All patients treated with VPR and only during the time of VPR treatment; for patients who switched from UPA to VPR, the period of UPA treatment is excluded. AE, adverse event; ASAT, aspartate aminotransferase; DB, double-blind; MedDRA, Medical Dictionary for Regulatory Activities; OL, open label; B-OL, blinded-to–open-label transition; TEAE, treatment-emergent adverse event; UPA, ulipristal acetate; VPR, vilaprisan. A summary of AEs and TEAEs by intensity is provided in Table S4. Among the total vilaprisan-treated group, study drug-related TEAEs were reported as mild in intensity in 120 (18.5%) women, moderate in 81 (12.5%) women, and severe in 16 (2.5%) women. This observation was driven mostly by the two largest treatment groups (i.e., VPR-3/1 and VPR-6/2).

## Data Availability

Availability of the data underlying this publication will be determined according to Bayer’s commitment to the EFPIA/PhRMA “Principles for responsible clinical trial data sharing.” This pertains to the scope, timepoint, and process of data access. As such, Bayer commits to sharing (upon request from qualified scientific and medical researchers), patient-level clinical trial data, study-level clinical trial data, and protocols from clinical trials in patients for medicines and indications approved in the United States (US) and European Union (EU), as necessary for conducting legitimate research. This applies to data on new medicines and indications that have been approved by the EU and US regulatory agencies on or after 1 January 2014. Interested researchers can use https://vivli.org/ourmember/bayer/to request access to anonymized patient-level data and supporting documents from clinical studies to conduct further research that can help to advance medical science or to improve patient care. Information on the Bayer criteria for listing studies and other relevant information is provided in the Study Sponsors section of the portal. Data access will be granted to anonymized patient-level data, protocols, and clinical study reports after approval by an independent scientific review panel. Bayer is not involved in the decisions made by the independent review panel. Bayer will take all necessary measures to ensure that patient privacy is safeguarded.

## Supplementary Materials

The following supporting information can be downloaded at: https://www.mdpi.com/article/10.3390/jcm15093246/s1, Figure S1: Study design overview after implementation of protocol amendment due to label change for UPA; Figure S2: Percent change in the volume of the largest fibroid from baseline for the VPR-3/2 and UPA-3/2 arms (as measured by ultrasound); Table S1: Safety endpoints; Table S2: Amenorrhea rates during the last 28 days by treatment period (FAS); Table S3: PGI-S ratings for TP1 and TP2; Table S4 Summary of adverse events and treatment-emergent adverse events by maximum intensity.

## Abbreviations

The following abbreviations are used in this manuscript:

AE	Adverse events
AESI	Adverse events of special interest
ALT	Alanine transferase
ANCOVA	Analysis of covariance
AST	Aspartate aminotransferase
CI	Confidence interval
FAS	Full analysis set
GnRH	Gonadotropin-releasing hormone
HMB	Heavy menstrual bleeding
HRQoL	Health-related quality of life
MBL	Menstrual blood loss
MCID	Minimal clinically important difference
MRI	Magnetic resonance imaging
PGI-S	Patient Global Impression of Severity
PRAC	Pharmacovigilance Risk Assessment Committee
PROs	Patient-reported outcomes
PPS	Per-protocol set
QoL	Quality of life
SAE	Serious adverse event
SAF	Safety analysis set
SD	Standard deviation
SPRM	Selective progesterone receptor modulator
SS	Symptom severity
TEAE	Treatment-emergent adverse event
TP	Treatment period
UF	Uterine fibroid
UF-DBD	Uterine Fibroid Daily Bleeding Diary
UF-DSD	Uterine Fibroid Daily Symptom Diary
UFS-QoL	Uterine Fibroid Symptom and Quality of Life questionnaire
ULN	Upper limit of normal
UPA	Ulipristal acetate
VPR	Vilaprisan
