# Supplementary material for: Efficacy and Safety of Vilaprisan in the Treatment of Uterine Fibroids: Data from ASTEROID 5, a Phase 3 Multicenter Randomized Controlled Trial
Source: J Clin Med. 2026 Apr 24;15(9):3246. doi: 10.3390/jcm15093246 (PMC13164208; doi:10.3390/jcm15093246)
Supplement: Supplementary file 1 [file jcm-15-03246-s001.zip › ASTEROID5_Manuscript_Supplementary Materials_3Mar2026_tracked.pdf]

Supplementary Materials

Efficacy and safety of vilaprisan in the treatment of uterine fibroids: Data from ASTEROID 5, a phase 3 multicenter randomized controlled trial

K Gemzell-Danielsson[1], C-H Cho[2], P Vadász[3], R Wenzl[4], L Dong[5], T Faustmann[5], E Groettrup-Wolfers[5], K Laapas[6], S Parke[5], C Haberland[5], C Seitz[5,7*]

[1]Department of Women's and Children's Health, Karolinska Institutet, and Karolinska University Hospital, Stockholm 171 77, Sweden; [2]Department of Obstetrics and Gynecology, School of Medicine, Keimyung University, Daegu 42601, Republic of Korea; [3]Department of Obstetrics and Gynecology, Selye János Hospital, Komárom 2900, Hungary; [4]Department of Obstetrics and Gynecology, Medical University of Vienna, Vienna 1090, Austria; [5]Bayer AG, Berlin 13353, Germany; [6]Bayer Oy, Espoo 02100, Finland; [7]Institute of Clinical Pharmacology and Toxicology, Charité – Universitätsmedizin Berlin, Berlin 10117, Germany

Figure S1 Study design overview after implementation of protocol amendment due to label change for UPA. [a]Women who were receiving study treatment before the protocol amendment were allowed to continue into the open-label phase, and women recently randomized to the VPR-3/2 or UPA-3/2 groups were to start study treatment with open-label VPR-3/2 for up to two treatment periods of 12 weeks (i.e., 3 months, 3×28 days) with two menstrual bleeding episodes after the first or second treatment period. This was equivalent to a second screening period, as women had to re-consent their participation in the study, be re-assessed for eligibility, and undergo relevant baseline assessments before starting the open-label phase.

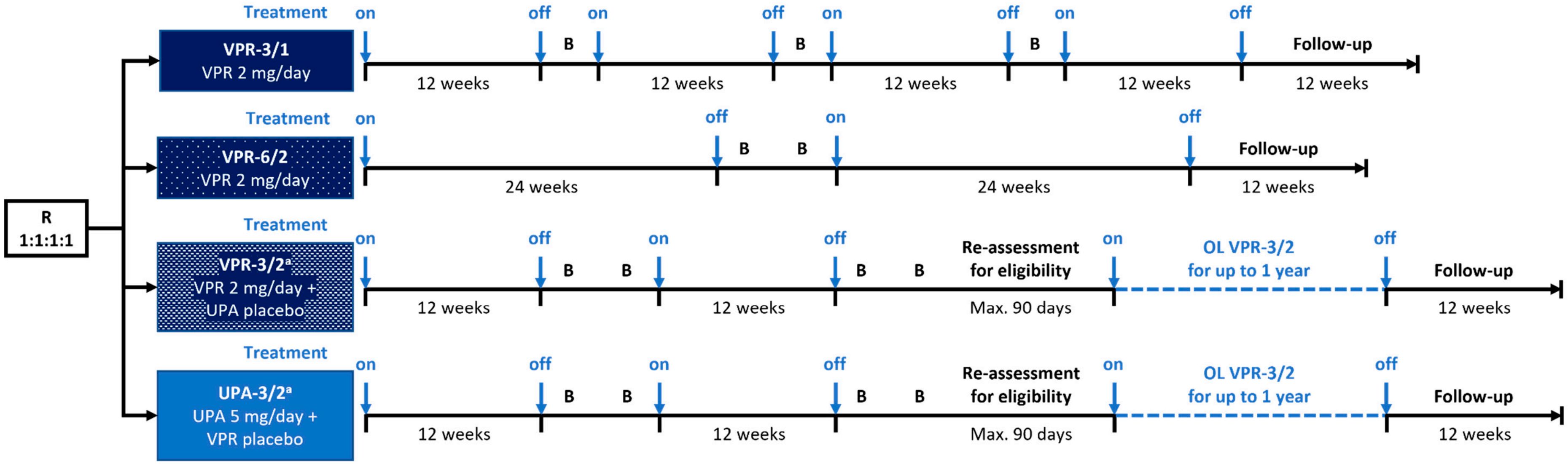

B, break, characterized as day(s) with bleeding/spotting of at least mild intensity of ≥1 day in duration, preceded and followed by ≥2 bleed-free days; off, treatment stopped; OL, open-label; on, treatment started; R, randomized; UPA, ulipristal acetate; VPR, vilaprisan

Figure S2 Percent change in the volume of the largest fibroid from baseline for the VPR-3/2 and UPA-3/2 arms (as measured by ultrasound).

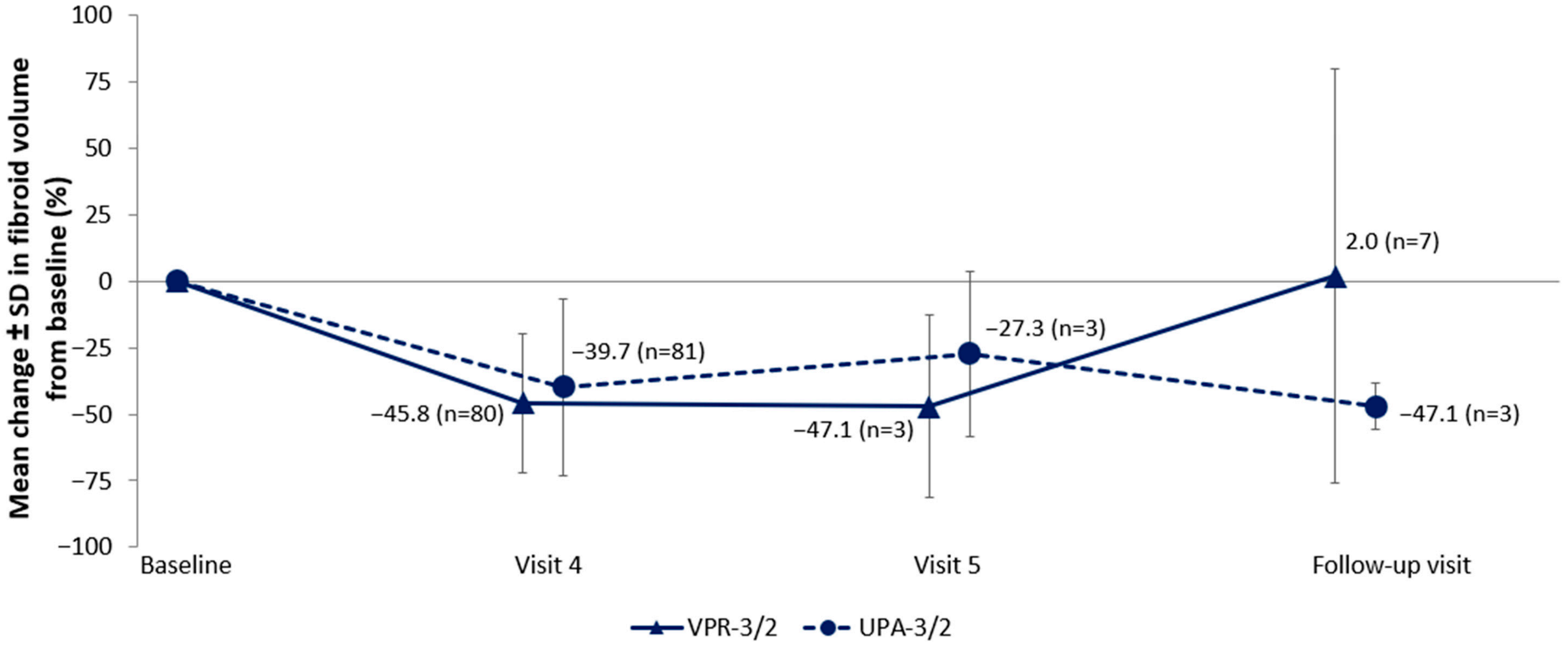

SD, standard deviation; UPA, ulipristal acetate; VPR, vilaprisan

Table S1 Safety endpoints.

Safety endpoints	
**Secondary safety endpoints**	**Other safety endpoints**
Endometrial histology (e.g., benign endometrium, presence or absence of hyperplasia[a] or malignancy)<br><br>Endometrial thickness	Endometrial histology (diagnosis of PAEC, individual features of PAEC)<br><br>Ovarian cysts (number, size)<br><br>Laboratory parameters<br><br>Adverse events<br><br>Cervical smear<br><br>Vital signs<br><br>UF-DBD bleeding pattern per 28 days and 84 days<br><br>Findings resulting from liver monitoring<br><br>Findings resulting from adrenal monitoring<br><br>Findings resulting from skin monitoring

[a]Hyperplasia was defined according to the World Health Organization 2014 criteria (Zaino, *et al.*, 2014). PAEC, progesterone receptor modulator-associated endometrial changes; UF-DBD, Uterine Fibroid Daily Bleeding Diary.

Table S2: Amenorrhea rates during the last 28 days by treatment period (FAS)

**Data are n (%) [95% CI]**	**Treatment group**			
Treatment period	VPR-3/1	VPR-6/2	VPR-3/2	UPA-3/2
TP1	222/271 (81.9) [76.8–86.3]	218/266 (82.0) [76.8–86.4]	80/90 (88.9) [80.5–94.5]	66/89 (74.2) [63.8–82.9]
TP2	182/217 (83.9) [78.3–88.5]	176/247 (71.3) [65.2–76.8]	2/3 (66.7) [9.4–99.2]	3/4 (75.0) [19.4–99.4]
TP3	111/121 (91.7) [85.3–96.0]	110/126 (87.3) [80.2–92.6]	—	—
TP4	39/48 (81.3) [67.4–91.1]	60/95 (63.2) [52.6–72.8]	—	—
TP1-OL	—	—	15/16 (93.8) [69.8–99.8]	20/22 (90.9) [70.8–98.9]

The 95% confidence intervals were calculated using the Clopper–Pearson method.

Table S3 PGI-S ratings for TP1 and TP2.

Data are n (%)		Treatment arm				
**Timepoint**	**PGI-S rating**	**VPR-3/1**	**VPR-6/2**	**VPR-3/2**	**UPA-3/2**	**Total VPR-treated[a]**
**Baseline**	**n**	**133 (100.0)**	**133 (100.0)**	**54 (100.0)**	**47 (100.0)**	**320 (100.0)**
	None	9 (6.8)	5 (3.8)	4 (7.4)	2 (4.3)	18 (5.6)
	Very mild	11 (8.3)	14 (10.5)	3 (5.6)	2 (4.3)	28 (8.8)
	Mild	19 (14.3)	25 (18.8)	5 (9.3)	9 (19.1)	49 (15.3)
	Moderate	63 (47.4)	46 (34.6)	20 (37.0)	17 (36.2)	129 (40.3)
	Severe	25 (18.8)	36 (27.1)	16 (29.6)	11 (23.4)	77 (24.1)
	Very severe	6 (4.5)	7 (5.3)	6 (11.1)	6 (12.8)	19 (5.9)
**First 28-day period TP1**	**n**	**157 (100.0)**	**159 (100.0)**	**55 (100.0)**	**53 (100.0)**	**371 (100.0)**
	None	15 (9.6)	14 (8.8)	6 (10.9)	5 (9.4)	35 (9.4)
	Very mild	30 (19.1)	21 (13.2)	9 (16.4)	5 (9.4)	60 (16.2)
	Mild	33 (21.0)	31 (19.5)	9 (16.4)	11 (20.8)	73 (19.7)
	Moderate	44 (28.0)	53 (33.3)	13 (23.6)	18 (34.0)	110 (29.6)
	Severe	29 (18.5)	30 (18.9)	14 (25.5)	12 (22.6)	73 (19.7)
	Very severe	6 (3.8)	10 (6.3)	4 (7.3)	2 (3.8)	20 (5.4)
**Second 28-day period TP1**	**n**	**153 (100.0)**	**151 (100.0)**	**55 (100.0)**	**55 (100.0)**	**359 (100.0)**
	None	28 (18.3)	17 (11.3)	12 (21.8)	8 (14.5)	57 (15.9)
	Very mild	32 (20.9)	34 (22.5)	10 (18.2)	17 (30.9)	76 (21.2)
	Mild	40 (26.1)	45 (29.8)	14 (25.5)	11 (20.0)	99 (27.6)
	Moderate	40 (26.1)	40 (26.5)	13 (23.6)	16 (29.1)	93 (25.9)
	Severe	10 (6.5)	13 (8.6)	4 (7.3)	2 (3.6)	27 (7.5)

	Very severe	3 (2.0)	2 (1.3)	2 (3.6)	1 (1.8)	7 (1.9)
**Third 28-day period TP1**	**n**	**165 (100.0)**	**155 (100.0)**	**59 (100.0)**	**51 (100.0)**	**379 (100.0)**
	None	31 (18.8)	16 (10.3)	18 (30.5)	9 (17.6)	65 (17.2)
	Very mild	45 (27.3)	44 (28.4)	12 (20.3)	19 (37.3)	101 (26.6)
	Mild	42 (25.5)	45 (29.0)	17 (28.8)	6 (11.8)	104 (27.4)
	Moderate	34 (20.6)	40 (25.8)	11 (18.6)	10 (19.6)	85 (22.4)
	Severe	12 (7.3)	5 (3.2)	0	6 (11.8)	17 (4.5)
	Very severe	1 (0.6)	5 (3.2)	1 (1.7)	1 (2.0)	7 (1.8)
**Fourth 28-day period TP1**	**n**	**4 (100.0)**	**-**	**1 (100.0)**	**1 (100.0)**	**5 (100.0)**
	None	0		1 (100.0)	0	1 (20.0)
	Very mild	2 (50.0)		0	0	2 (40.0)
	Mild	1 (25.0)		0	1 (100.0)	1 (20.0)
	Moderate	1 (25.0)		0	0	1 (20.0)
	Severe	0		0	0	0
	Very severe	0		0	0	0
**First 28-day period TP2**	**n**	**148 (100.0)**	**163 (100.0)**	**3 (100.0)**	**2 (100.0)**	**314 (100.0)**
	None	24 (16.2)	33 (20.2)	1 (33.3)	0	58 (18.5)
	Very mild	42 (28.4)	49 (30.1)	1 (33.3)	0	92 (29.3)
	Mild	42 (28.4)	47 (28.8)	0	0	89 (28.3)
	Moderate	27 (18.2)	22 (13.5)	1 (33.3)	1 (50.0)	50 (15.9)
	Severe	11 (7.4)	9 (5.5)	0	1 (50.0)	20 (6.4)
	Very severe	2 (1.4)	3 (1.8)	0	0	5 (1.6)
**Second 28-day period TP2**	**n**	**135 (100.0)**	**154 (100.0)**	**3 (100.0)**	**2 (100.0)**	**292 (100.0)**

	None	37 (27.4)	33 (21.4)	2 (66.7)	1 (50.0)	72 (24.7)
	Very mild	45 (33.3)	47 (30.5)	0	1 (50.0)	92 (31.5)
	Mild	29 (21.5)	37 (24.0)	1 (33.3)	0	67 (22.9)
	Moderate	15 (11.1)	28 (18.2)	0	0	43 (14.7)
	Severe	6 (4.4)	8 (5.2)	0	0	14 (4.8)
	Very severe	3 (2.2)	1 (0.6)	0	0	4 (1.4)
**Third 28-day period TP2**	**n**	**137 (100.0)**	**149 (100.0)**	**3 (100.0)**	**3 (100.0)**	**289 (100.0)**
	None	31 (22.6)	36 (24.2)	2 (66.7)	1 (33.3)	69 (23.9)
	Very mild	46 (33.6)	47 (31.5)	0	1 (33.3)	93 (32.2)
	Mild	34 (24.8)	28 (18.8)	0	0	62 (21.5)
	Moderate	16 (11.7)	28 (18.8)	0	0	44 (15.2)
	Severe	8 (5.8)	8 (5.4)	1 (33.3)	1 (33.3)	17 (5.9)
	Very severe	2 (1.5)	2 (1.3)	0	0	4 (1.4)
**Fourth 28-day period TP2**	**n**	**1 (100.0)**	**3 (100.0)**	**-**	**-**	**4 (100.0)**
	None	0	0			0
	Very mild	1 (100.0)	2 (66.7)			3 (75.0)
	Mild	0	0			0
	Moderate	0	1 (33.3)			1 (25.0)
	Severe	0	0			0
	Very severe	0	0			0

[a]All patients treated with VPR and only during the time with VPR treatment; for patients who switched from UPA to VPR, the period of UPA treatment is excluded. PGI-S, Patient Global Impression of Severity; TP, treatment period; UPA, ulipristal acetate; VPR, vilaprisan.

Table S4 Summary of adverse events and treatment-emergent adverse events by maximum intensity.

**Data are n (%)**	**Treatment group**						
Type of AE	VPR-3/1<br>N=271	VPR-6/2<br>N=266	VPR-3/2 (DB)<br>N=86	UPA-3/2 (DB)<br>N=89	VPR-3/2 (OL)<br>N=19	VPR-3/2 (B-OL)<br>N=23	**Total VPR-treated[a]**<br>**N=649**
Any AE	228 (84.1)	232 (87.2)	66 (76.7)	70 (78.7)	15 (78.9)	15 (65.2)	548 (84.4)
Mild	98 (36.2)	95 (35.7)	26 (30.2)	27 (30.3)	7 (36.8)	7 (30.4)	228 (35.1)
Moderate	89 (32.8)	104 (39.1)	26 (30.2)	32 (36.0)	8 (42.1)	6 (26.1)	230 (35.4)
Severe	41 (15.1)	33 (12.4)	14 (16.3)	11 (12.4)	0	2 (8.7)	90 (13.9)
Any study-drug-related AE	97 (35.8)	112 (42.1)	17 (19.8)	30 (33.7)	4 (21.1)	7 (30.4)	235 (36.2)
Mild	57 (21.0)	60 (22.6)	9 (10.5)	16 (18.0)	3 (15.8)	4 (17.4)	131 (20.2)
Moderate	33 (12.2)	45 (16.9)	6 (7.0)	11 (12.4)	1 (5.3)	3 (13.0)	88 (13.6)
Severe	7 (2.6)	7 (2.6)	2 (2.3)	3 (3.4)	0	0	16 (2.5)
Any AE related to procedures required by the protocol	19 (7.0)	24 (9.0)	1 (1.2)	4 (4.5)	0	0	44 (6.8)
Any AE leading to study drug discontinuation	23 (8.5)	20 (7.5)	3 (3.5)	6 (6.7)	0	0	46 (7.1)
Any AESI	90 (33.2)	101 (38.0)	19 (22.1)	27 (30.3)	3 (15.8)	4 (17.4)	216 (33.3)
Any SAE	64 (23.6)	55 (20.7)	10 (11.6)	17 (19.1)	3 (15.8)	5 (21.7)	137 (21.1)
Any study drug-related SAE	9 (3.3)	5 (1.9)	0	1 (1.1)	0	1 (4.3)	15 (2.3)
Any SAE leading to study drug discontinuation	1 (0.4)	4 (1.5)	0	1 (1.1)	0	0	5 (0.8)
Any TEAE	190 (70.1)	184 (69.2)	39 (45.3)	48 (53.9)	9 (47.4)	9 (39.1)	427 (65.8)

Maximum intensity for any TEAE							
Mild	106 (39.1)	97 (36.5)	23 (26.7)	26 (29.2)	6 (31.6)	5 (21.7)	234 (36.1)
Moderate	64 (23.6)	71 (26.7)	13 (15.1)	18 (20.2)	3 (15.8)	4 (17.4)	154 (23.7)
Severe	20 (7.4)	16 (6.0)	3 (3.5)	4 (4.5)	0	0	39 (6.0)
Any study-drug-related TEAE	91 (33.6)	105 (39.5)	16 (18.6)	29 (32.6)	3 (15.8)	4 (17.4)	217 (33.4)
Maximum intensity for any study-drug-related TEAE							
Mild	52 (19.2)	57 (21.4)	8 (9.3)	15 (16.9)	3 (15.8)	2 (8.7)	120 (18.5)
Moderate	32 (11.8)	41 (15.4)	6 (7.0)	12 (13.5)	0	2 (8.7)	81 (12.5)
Severe	7 (2.6)	7 (2.6)	2 (2.3)	2 (2.2)	0	0	16 (2.5)

[a]All patients treated with VPR and only during the time with VPR treatment; for patients who switched from UPA to VPR, the period of UPA treatment is excluded. AE, adverse event; AESI, adverse event of special interest; B, blind; DB, double blind; OL, open label; SAE, serious adverse event; TEAE, treatment-emergent adverse event; UPA, ulipristal acetate; VPR, vilaprisan.
